# Climate change perception–impact–adaptation pathways among farmers: evidence from seven major agricultural governorates in Egypt

**DOI:** 10.1038/s41598-026-63674-x

**Published:** 2026-07-29

**Authors:** Eman S. Swelam, Moataz S. Abdallah, Hamed A. Ead, Attia M. El-Tantawi, Fatma S. Ahmed

**Affiliations:** 1 Department of Economic Entomology and Pesticides, Faculty of Agriculture, Cairo University, Giza, 12613 Egypt; 2 Faculty of Arts, Cairo University, Giza, Egypt; 3 Faculty of Science, Cairo University, Giza, Egypt; 4 Faculty of African Postgraduate Studies, Cairo University, Giza, Egypt

**Keywords:** Climate change perception, Composite indices, Adaptation practices, Institutional support, Modern irrigation systems, Structural constraints, Egyptian agriculture, Climate sciences, Environmental social sciences

## Abstract

Climate change poses growing challenges to Egyptian agriculture, yet farmers’ adaptive responses remain uneven. This study examines perceptions of climate change, its impacts, and adaptation pathways among 2953 farmers across seven major agricultural governorates in Egypt. Utilizing composite indices, this study captures the multidimensionality of climate awareness, perceived impacts, adaptation practices, barriers, and institutional support. The findings reveal a clear mismatch between high levels of climate awareness and the limited uptake of technical adaptation measures. Farmers’ responses are largely concentrated on low-cost, incremental practices, while capital-intensive technologies, such as modern irrigation systems and digital tools, are rarely adopted. Traditional irrigation methods continue to dominate, despite widespread reports of climate-related impacts on agriculture, including severe productivity declines in key crops. Statistical analyses show a strong positive association between adaptation practices and reported changes in productivity, whereas the perceived severity of climate impacts is not significantly associated with adaptation levels. These results indicate that farmers’ adaptation decisions are primarily reactive—shaped by tangible production outcomes and structural constraints rather than by awareness alone. These findings underscore the importance of complementing awareness-focused interventions with targeted financial, market, and extension support to enable effective, transformative climate adaptation in Egyptian agriculture.

**Supplementary Information:**

The online version contains supplementary material available at 10.1038/s41598-026-63674-x.

## Introduction

Climate change poses a severe threat to global agricultural systems, threatening long-term gains in food production and rural livelihoods^1,2^. Increasing frequencies of droughts, floods, and heatwaves are disrupting crop cycles and reducing yields across diverse regions^3–5^. According to the Intergovernmental Panel on Climate Change (IPCC), climate-related extremes have already reduced agricultural productivity, particularly through drought and heat stress affecting staple crops^6^. Quantitative assessments further suggest that global crop yields could decline by 3–12% by mid-century under continued climate change^7^.

These impacts are not evenly distributed. Smallholder farmers in developing regions face disproportionate risks due to limited financial, technical, and institutional capacity to respond effectively^8–10^. Evidence from comparable smallholder systems shows that risk perception, information access, extension services, land tenure, income diversification, and farmer organizations strongly shape farmers’ capacity to adopt climate-risk management and conservation practices^10,11^. As a result, climate change represents an immediate and ongoing stressor for agricultural livelihoods rather than a distant future concern. This context highlights the importance of understanding how farmers perceive climate change, experience its impacts, and translate awareness into adaptive responses.

Egypt represents a particularly vulnerable case. Agriculture provides livelihoods for more than half of the population and remains central to food security and rural employment^12^. At the same time, the country’s farming systems operate under severe natural constraints. Over 94% of Egypt’s freshwater supply originates from the River Nile, making agricultural production highly sensitive to hydrological disruptions^13–15^.

Climate change intensifies these pressures through regionally distinct mechanisms. In the Nile Delta, sea-level rise has accelerated saltwater intrusion and soil salinization, with reported crop losses reaching 30–40% in some seasons^16,17^. In Upper Egypt, rising temperatures and increasing water scarcity threaten agricultural livelihoods, particularly among rural households that depend heavily on farming income^18^.

At the farm level, Egyptian farmers have increasingly reported changes in weather patterns, pest prevalence, and crop performance consistent with climate projections^19,20^. Warmer winter temperatures have enabled pests and pathogens to persist and spread, intensifying production challenges^21,22^. For example, studies on potato and tomato production indicate that elevated temperatures have contributed to more frequent late blight outbreaks, increasing reliance on fungicides^23^. These localized impacts illustrate how climatic changes translate into tangible agricultural stressors affecting day-to-day farm management decisions.

Farmers’ awareness and understanding of climate change play a critical role in shaping their responses to these challenges^24^. Earlier studies suggested relatively low awareness among Egyptian farmers^25^, while more recent research indicates that awareness has increased. However, growing awareness does not consistently lead to the adoption of effective technical adaptation measures, reflecting what has been described as an “attitude–action gap”^26^.

In practice, farmers have adopted a range of autonomous adaptation strategies, including adjustments in planting dates and the use of heat-tolerant crop varieties^27^. While these measures can reduce short-term exposure to climate stress, they are often incremental and reactive. More transformative adaptations—such as modern irrigation systems or advanced pest management technologies—remain less accessible due to financial, technical, and institutional constraints^28^.

Against this backdrop, this study examines perceptions of climate change, its impacts, and adaptation pathways among Egyptian farmers across seven major agricultural governorates. While previous studies have highlighted the vulnerability of Egyptian agriculture, most have focused on isolated geographic areas or singular adaptation practices. This study distinguishes itself by leveraging a large, multi-governorate dataset to capture a comprehensive, national-scale perspective. Furthermore, the novelty of this research lies in its use of composite indices to quantitatively model the 'perception–impact–adaptation’ pathway. By identifying the specific structural breakpoints where high awareness fails to translate into action, this study provides highly targeted, empirical evidence to inform and refine national climate adaptation policies.

## Results

### Socio-demographic characteristics

The farmers surveyed (N = 2953) represented a diverse demographic profile (Table 1). Most respondents were aged 46–60 years (37.9%), followed by those aged 30–45 (27.7%). Approximately one-third (33.7%) held a university degree or higher, while 13.1% were illiterate. Farming experience exceeded 16 years for the majority (63.2%), and farming was the main source of income for 38% of participants. Family involvement in farming was common, with 40.3% reporting 3–5 family members engaged. Land tenure was mixed: 37.7% both owned and rented land, while 35.1% relied exclusively on renting.

**Table 1:** Socio-demographic characteristics of surveyed farmers (N = 2953).

Characteristic	Category	Frequency (n)	Percentage (%)	95% CIs*
Age group	< 30 years	539	18.3	16.9–19.7
	30–45 years	817	27.7	26.1–29.3
	46–60 years	1120	37.9	36.2–39.7
	> 60 years	477	16.2	14.9–17.5
Education level	Illiterate	388	13.1	12.0–14.4
	Primary	726	24.6	23.1–26.2
	Secondary	844	28.6	27.0–30.2
	University or higher	995	33.7	32.0–35.4
Years of farming experience	< 5 years	225	7.6	6.69–8.64
	5–15 years	860	29.1	27.5–30.8
	16–30 years	969	32.8	31.1–34.5
	> 30 years	899	30.4	28.8–32.1
Farming as main income	Main source	1122	38.0	36.3–39.8
	Secondary source	1831	62.0	60.2–63.7
Family members involved in farming	1–2 members	95	3.2	2.61–3.92
	3–5 members	1189	40.3	38.5–42.0
	> 5 members	894	30.3	28.6–32.0
	0 (farmer only)	775	26.2	24.7–27.9
Land tenure status	Own	744	25.2	23.7–26.8
	Rent	1037	35.1	33.4–36.9
	Mix (own & rent)	1112	37.7	35.9–39.4
	Worker only	60	2.0	1.55–2.61

CIs = Confidence Intervals.

### General farming practices

Agricultural modernization remains constrained among the surveyed population. Traditional flood irrigation was the dominant system (75%), with only 13.7% and 10.9% using drip or sprinkler systems, respectively (Table 2). Most respondents utilized mixed chemical–organic fertilizers (84.6%) and relied on partial mechanization (52.3%). Crop rotation is a nearly universal practice (96.6%), and improved seed varieties are widely adopted (72.9%), sourced primarily through the Ministry of Agriculture (58.2%).

**Table 2:** General farming practices of respondents (N = 2953).

Practice	Category	Frequency (n)	Percentage (%)	95% CIs*
Main irrigation system	Traditional flood	2214	75.0	73.4–76.5
	Drip	406	13.7	12.6–15.0
	Sprinkler / Pivot	321	10.9	9.77–12.0
	Mixed modern systems	12	0.4	0.21–0.71
Main water source	Canal	1194	40.4	38.7–42.2
	River Nile	369	12.5	11.4–13.7
	Underground wells	448	15.2	13.9–16.5
	Treated wastewater	175	5.9	5.10–6.84
	Mixed sources	767	26.0	24.4–27.6
Fertilizer type	Chemical	405	13.7	12.5–15.0
	Organic	50	1.7	1.26–2.23
	Mixed (chemical + organic)	2498	84.6	83.2–85.8
Mechanization	Fully mechanized	1128	38.2	36.5–40.0
	Partially mechanized	1545	52.3	50.5–54.1
	Not mechanized	280	9.5	8.45–10.6
Crop rotation	Yes	2853	96.6	95.9–97.2
	No	100	3.4	2.76–4.10
Seed type used	Improved	2154	72.9	71.3–74.5
	Local/traditional	391	13.2	12.1–14.5
	Hybrid	408	13.8	12.6–15.1
Seed source	Ministry of Agriculture	1718	58.2	56.4–59.9
	NGOs / private sector	1235	41.8	40.1–43.6

CIs = Confidence Intervals.

### Climate change perceptions

**Fig. 1:**
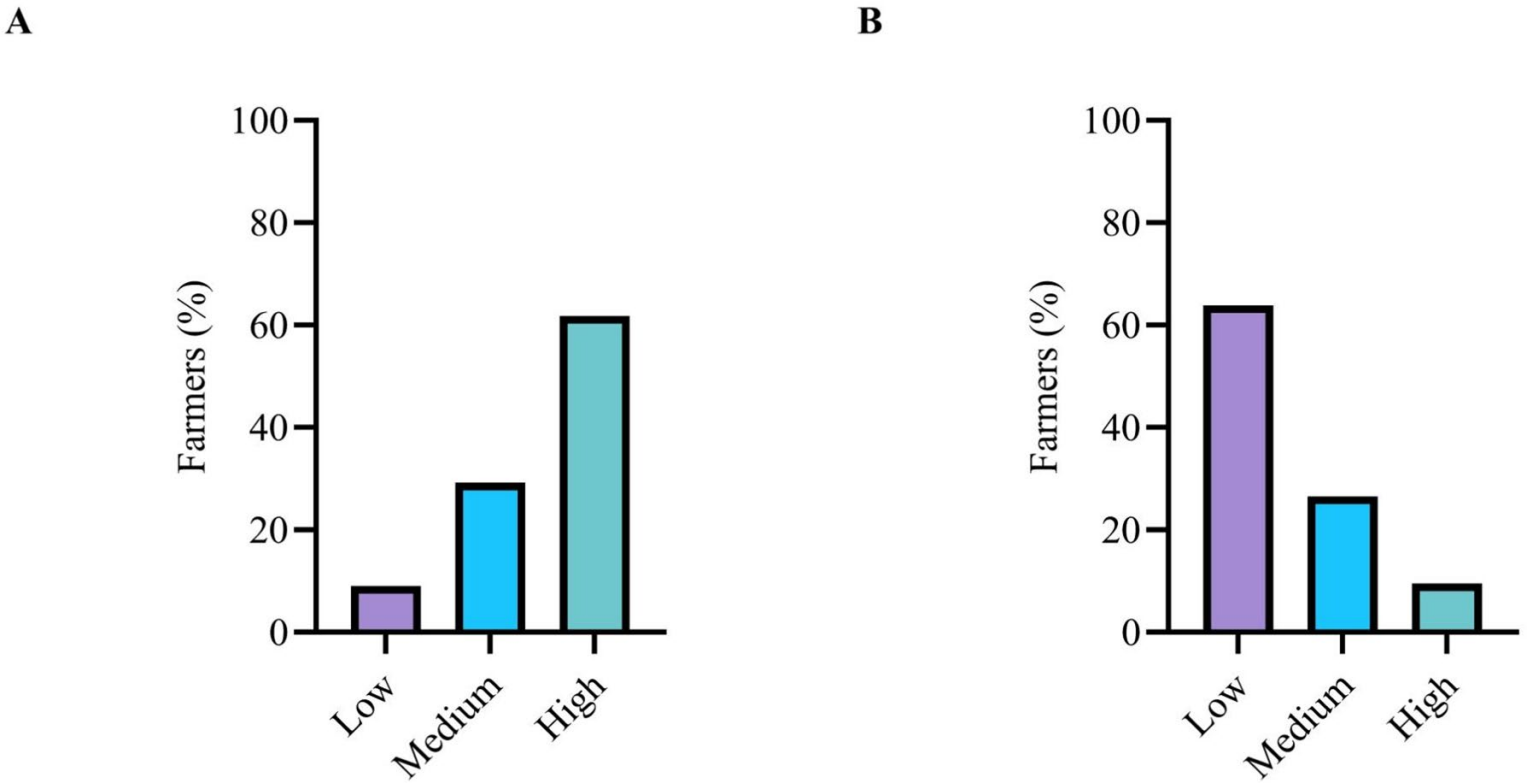
Proportion of farmers classified into low, medium, and high for (**A**) the climate awareness index (CAI) and (**B**) the climate impact severity index (CISI) (N = 2953).

Farmers exhibited high baseline awareness of climate-induced environmental shifts. As detailed in Table 3, 61.8% of respondents were classified in the high-awareness category of the Climate Awareness Index (CAI) (Fig. 1A). However, formal institutional guidance is lacking; the vast majority rely on peer-to-peer knowledge sharing (47.1%) and the media (25.1%), with only 3.6% receiving climate-specific guidance from governmental extension services.

**Table 3:** Climate change perceptions among farmers (n = 2953).

Variable	Category	Frequency (n)	Percentage (%)	95% CIs*
Awareness of climate change impacts	Very high	1121	38.0	36.2–39.7
	High	704	23.8	22.3–25.4
	Medium	863	29.2	27.6–30.9
	Low	265	9.0	7.97–10.1
Source of climate information	Media	741	25.1	23.6–26.7
	Governmental extension	105	3.6	2.92–4.29
	Other farmers	1390	47.1	45.3–48.9
	Not searching	717	24.3	22.8–25.9
Change in rainfall pattern	Significant increase	2116	71.7	70.0–73.3
	Slight increase	729	24.7	23.2–26.3
	No change	87	2.9	2.37–3.62
	Slight decrease	21	0.7	0.44–1.08
Change in temperature	Significant increase	2031	68.8	67.1–70.4
	Slight increase	640	21.7	20.2–23.2
	No change	282	9.5	8.51–10.7
Seasonal changes	Late start of the season	1418	48.0	46.2–49.8
	Early end of season	252	8.5	7.55–9.60
	No change	39	1.3	0.94–1.80
	Transitional periods	1244	42.1	40.4–43.9
Extreme weather events	Significant increase	1914	64.8	63.1–66.5
	Slight increase	739	25.0	23.5–26.6
	No change	297	10.1	9.0–11.2
	Slight decrease	3	0.1	0.00–0.30

Response categories with a frequency of 0 are not shown. CIs = Confidence Intervals.

Perceptions of environmental shifts strongly align with regional climate projections. A substantial majority reported significant increases in rainfall variability (71.7%), rising temperatures (68.8%), and extreme weather events (64.8%). Seasonal disruptions were similarly widespread, with 48.0% observing a late start to the agricultural season. Despite these high individual perception metrics, the aggregated Climate Impact Severity Index (CISI) indicates that 63.9% of farmers fall into the low severity category (Fig. 1B). This highlights a nuance in perception: while farmers recognize discrete climatic shifts, the cumulative perceived severity of these events remains moderate for the majority.

### Climate impacts on agriculture

**Fig. 2:**
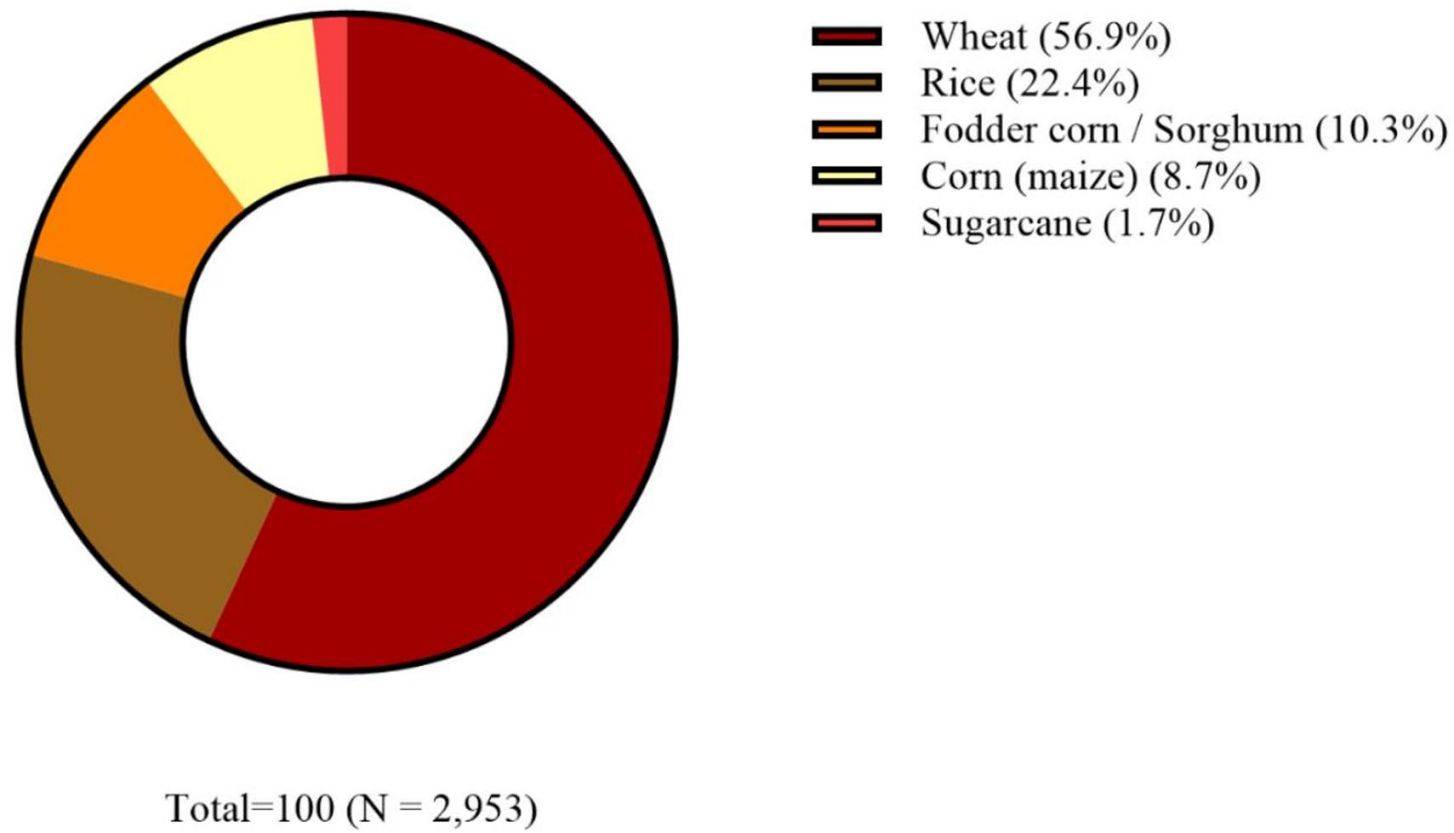
Distribution of the most heavily affected crops due to climate impacts, as reported by farmers (N = 2953).

The tangible consequences of climate change on farm outcomes are severe (Table 4). Productivity declines are widespread, with 53.1% reporting a significant decrease. Furthermore, 84.2% of farmers observed a significant escalation in pest incidence. These biological pressures have forced reactive shifts in agricultural planning, leading 14.6% of farmers to completely abandon specific crops due to pest infestations. Wheat emerged as the most highly vulnerable crop (56.9%), followed by rice (22.4%) and fodder corn/sorghum (10.3%) (Fig. 2), underscoring a direct threat to Egypt’s staple food security.

**Table 4:** Climate Impacts on Agriculture (N = 2953).

Impact variable	Category	Frequency (n)	Percentage (%)	95% CIs*
Change in productivity	Significant decrease	1567	53.1	51.3–54.9
	Slight decrease	861	29.2	27.5–30.8
	No change	525	17.8	16.4–19.2
Change in pests	Significant increase	2487	84.2	82.9–85.5
	Slight increase	466	15.8	14.5–17.2
Crop change due to pests	Complete crop change	432	14.6	13.4–16.1
	Slight crop change	520	17.6	16.3–19.0
	No change	1881	63.7	61.9–65.4
	Do not know	120	4.1	3.38–4.84

CIs = Confidence Intervals.

### Adaptation practices

#### Adoption of individual adaptation practices

Farmers reported varying levels of adoption across adaptation practices (Table 5). Sustainable agricultural practices saw the highest adoption (49.4% full adoption). However, 21.7% reported no adoption, and 8.8% were unsure or lacked knowledge of these practices.

**Table 5:** Adoption of climate adaptation practices among farmers (N = 2953).

Adaptation practice	Category	Frequency (n)	Percent (%)	95% CIs*
Adoption of sustainable practices	Adopted completely	1,458	49.4	47.6–51.2
	Adopted some	593	20.1	18.7–21.6
	Not adopted (choice not to use)	642	21.7	20.3–23.3
	Do not know (lack of awareness)	260	8.8	7.81–9.88
Irrigation modernization	Improved completely	51	1.7	1.29–2.26
	Improved partially	553	18.7	17.3–20.2
	Still using old technology	2314	78.4	76.8–79.8
	Do not know how	35	1.2	0.85–1.64
Use of adapted varieties	Yes, completely	526	17.8	16.5–19.2
	Yes, sometimes	1044	35.4	33.6–37.1
	No, never	195	6.6	5.73–7.56
	Do not know them	1188	40.2	38.5–42.0
Pest/disease management	Use innovative methods (*e.g*., biological control)	39	1.3	0.94–1.80
	Rely solely on chemicals	2625	88.9	87.7–90.0
	Do not know	289	9.8	8.74–10.9
Use of digital tools	Used completely	114	3.9	3.19–4.62
	Used some	1387	47.0	45.2–48.8
	Not used	769	26.0	
	Do not know them	683	23.1	24.5–27.7
Collaboration with other farmers	Yes, regularly	499	16.9	15.6–18.3
	Yes, sometimes	2224	75.3	73.7–76.9
	No, never	95	3.2	2.61–3.92
	Do not know how	135	4.6	3.85–5.39

CIs = Confidence Intervals.

Irrigation modernization remained limited: only 1.7% had fully modernized their systems, while 18.7% had partially improved them. The vast majority (78.4%) continued to rely on traditional irrigation technologies, while 1.2% reported not knowing how to improve their systems.

The use of climate-adapted crop varieties was moderate, with 17.8% adopting them completely and 35.4% using them sometimes. Notably, a large proportion (40.2%) reported not knowing about adapted varieties, and 6.6% had never adopted them.

Pest and disease control showed the lowest level of innovation. Only 1.3% reported using innovative pest/disease management practices, while 88.9% relied solely on chemical methods.

Digital tools showed mixed adoption: while 3.9% used them fully and 47.0% used them partially, nearly half of the respondents either did not use them (26.0%) or were unfamiliar with them (23.1%). Collaboration among farmers was relatively common: 16.9% collaborated regularly and 75.3% occasionally. Only 3.2% never collaborated.

#### Adaptation practices index (API)

**Fig. 3:**
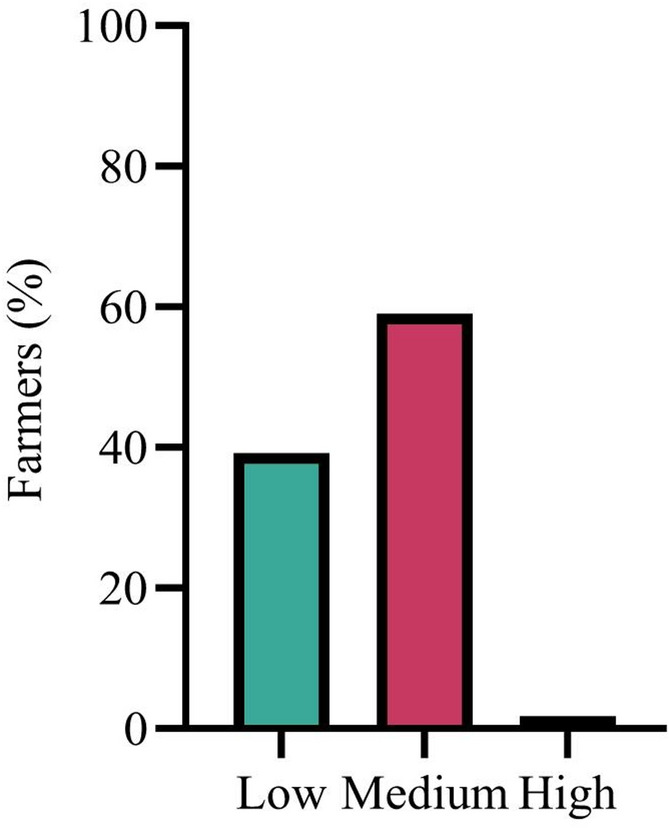
Distribution of farmers across Adaptation Practices Index (API) categories (N = 2953). Values represent the percentage of farmers classified into low, moderate, and high adaptation levels.

The aggregate API demonstrates a systemic lack of transformative adaptation. As illustrated in Fig. 3, only 1.8% of farmers achieved a high level of adaptation. The majority (59.0%) fall into the moderate category, engaging only in partial or low-cost adjustments, while 39.2% exhibit low adaptive capacity.

### Barriers to climate adaptation

The transition toward climate-smart agriculture is hindered by a complex web of structural constraints (Table 6). High input costs (26.1%) and water scarcity (21.8%) are dominant physical and economic barriers. Specifically, regarding sustainable practices, the prohibitive initial capital cost was cited as the primary obstacle by 49.3% of respondents, compounded by uncertainty regarding the effectiveness of these practices (33.6%).

**Table 6:** Barriers to climate adaptation among farmers (N = 2953).

Barrier variable	Category	Frequency (n)	Percent (%)	95% CIs*
Biggest challenge	Climate change impacts	975	33.0	31.3–34.7
	High input cost	772	26.1	24.6–27.8
	Water scarcity	643	21.8	20.3–23.3
	Pest & disease spread	484	16.4	15.1–17.8
	Lack of modern knowledge	79	2.7	2.12–3.32
Main barrier to sustainable practices	High initial cost	1456	49.3	47.5–51.1
	Uncertainty about effectiveness	993	33.6	31.9–35.4
	Lack of technical training	454	15.4	14.1–16.7
	Lack of governmental support	50	1.7	1.26–2.23
Difficulty obtaining tolerant seeds	No difficulty	1713	58.0	56.2–59.8
	Do not know	845	28.6	27.0–30.3
	Significant difficulty	246	8.3	7.36–9.39
	Some difficulty	149	5.0	4.28–5.90
Marketing difficulties	Always	1203	40.7	39.0–42.5
	Sometimes	675	22.9	21.4–24.4
	Rarely	300	10.2	9.09–11.3
	No	775	26.2	24.7–27.9
Economic impact	Very high impact	1952	66.1	64.4–67.8
	Moderate impact	761	25.8	24.2–27.4
	Low impact	240	8.1	7.17–9.17
Skilled labor shortage	Very high impact	1338	45.3	43.5–47.1
	Moderate impact	874	29.6	28.0–31.3
	Low impact	711	24.1	22.6–25.7
	No impact	30	1.0	0.69–1.45

CIs = Confidence Intervals.

**Fig. 4:**
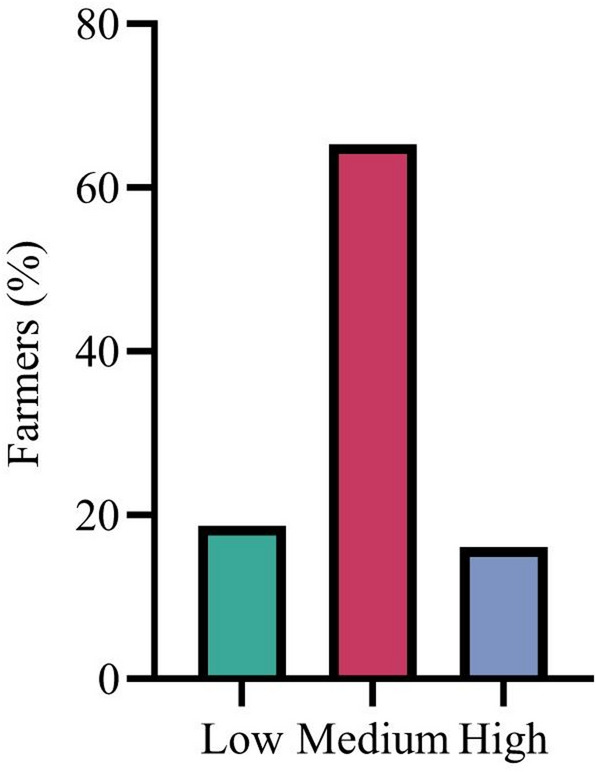
Distribution of Barrier Level Index (BAI) Categories Among Farmers.

These constraints translate into severe socioeconomic vulnerability. A vast majority (66.1%) reported experiencing very high economic impacts from climate disruptions, exacerbated by chronic marketing difficulties and skilled labor shortages. Consequently, the Barriers to Adaptation Index (BAI) (Fig. 4) confirms that most farmers operate within a moderate-to-high barrier environment.

### Support and training availability for climate adaptation

Institutional support systems are currently insufficient to meet farmer needs (Table 7). An information-poverty trap is evident: 48.3% of farmers have never received climate-related training, and 62.9% do not utilize weather forecasts. Yet, the demand for intervention is near-universal, with 84.6% declaring an urgent need for awareness and outreach programs. Farmers primarily desire political support (34.8%) and technical equipment/varieties (27.8%), with government extension services identified as the preferred delivery mechanism (46.4%).

**Table 7:** Support and training related to climate adaptation (N = 2953).

Support variable	Category	Frequency (n)	Percent (%)	95% CIs*
Training received	No training	1427	48.3	46.5–50.1
	Yes, sometimes	1136	38.5	36.7–40.2
	Do not know	378	12.8	11.6–14.1
	Yes, regular	12	0.4	0.21–0.71
Weather forecast use	No, never	1858	62.9	61.2–64.6
	Yes, regularly	640	21.7	20.2–23.2
	Yes, sometimes	371	12.6	11.4–13.8
	Do not know	84	2.8	2.28–3.51
Urgency of awareness need	Urgent need	2497	84.6	83.2–85.8
	Limited need	396	13.4	12.2–14.7
	No need	60	2.0	1.55–2.61
Type of support needed	Political support	1028	34.8	33.1–36.5
	Technical equipment/new varieties	821	27.8	26.2–29.4
	Technical training/extension	750	25.4	23.9–27.0
	Financial support	354	12.0	10.9–13.2
Preferred support source	Government extension	1371	46.4	44.6–48.2
	Farmers’ organizations	753	25.5	24.0–27.1
	Media & internet	445	15.1	13.8–16.4
	Private sector	228	7.7	6.78–8.74
	Other farmers	156	5.3	4.50–6.15

CIs = Confidence Intervals.

**Fig. 5:**
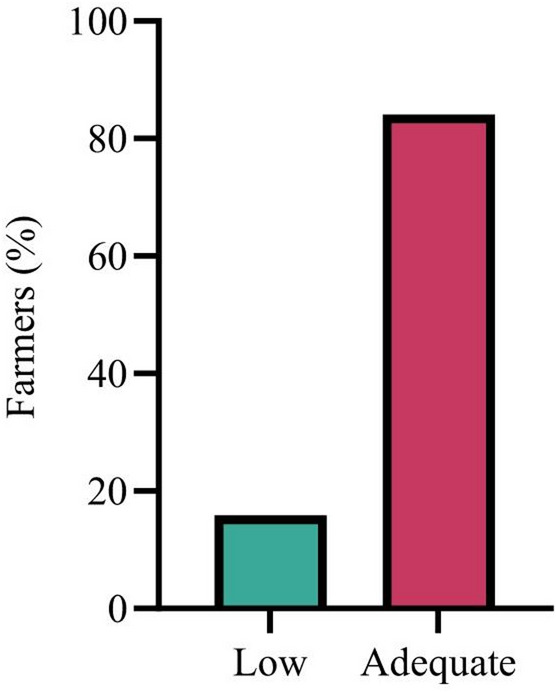
Distribution of Support and Training Index (STI) categories among farmers (N = 2953). STI was derived from training access and awareness-need measures.

The support and training index (STI) was derived from training access and awareness-need measures. Most farmers (84.1%) exhibited adequate support, while 15.9% had low support, highlighting substantial disparities in the availability of training and outreach (Fig. 5).

### Regional differences in awareness, impacts, adaptation, and barriers

**Fig. 6:**
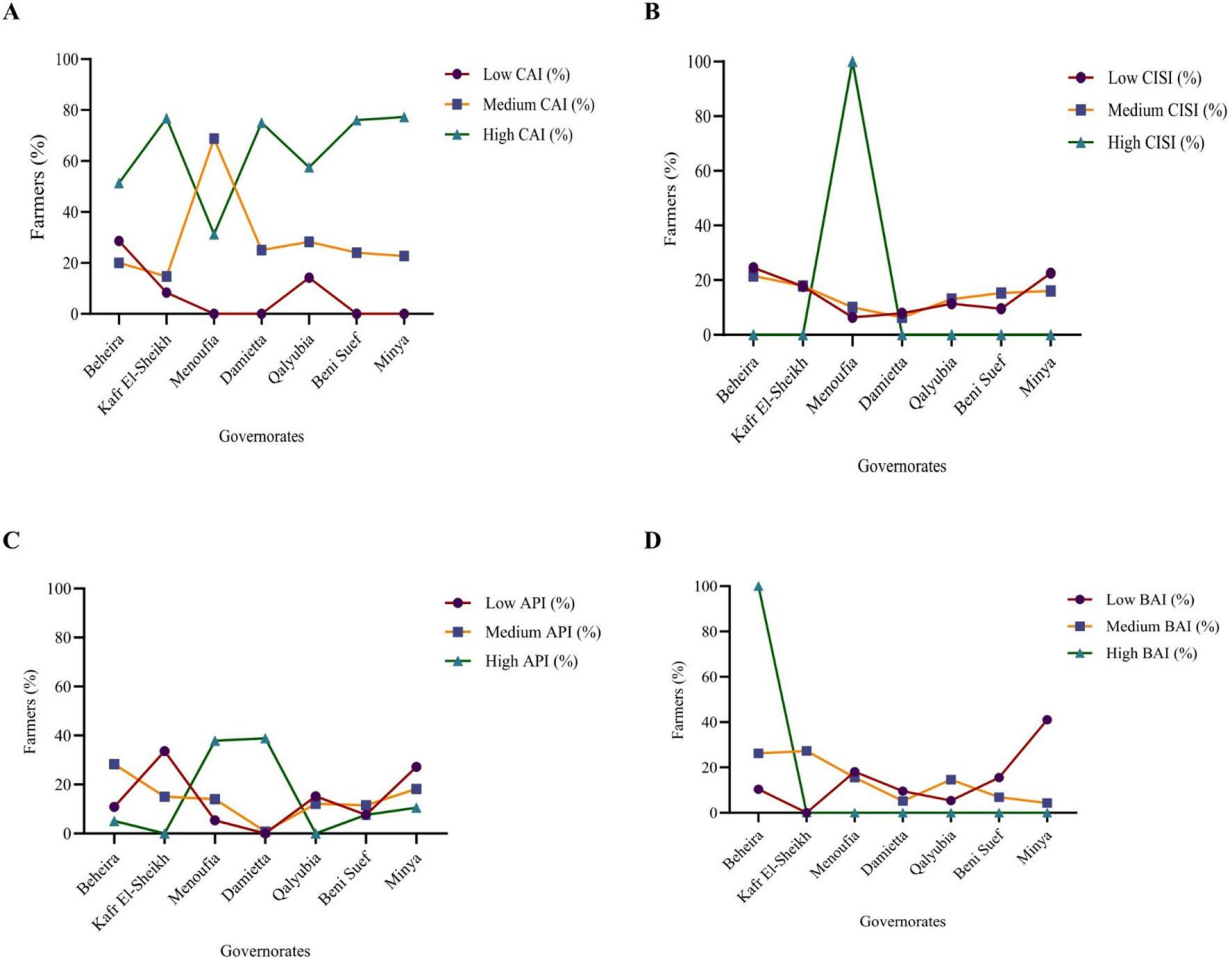
Regional distribution of Climate Awareness Index (CAI), Climate Impact Severity Index (CISI), Adaptation Practices Index (API), and Barrier Level Index (BAI) across seven Egyptian governorates. Each panel shows the percentage of farmers in the low, moderate, or high categories for the respective index.

Significant regional heterogeneity was observed across all climate-related indices (Table 8). Climate Awareness (CAI) varied considerably by governorate (χ^2^ = 876.2, *P* < 0.001). Beheira and Kafr El-Sheikh had higher proportions of farmers within the low-awareness group, whereas Menoufia and Minya showed markedly higher levels of climate awareness (Fig. 6A).

**Table 8:** Chi-square results for governorate comparisons across climate-related indices.

Index compared with the governorate	Χ^2^ (df)	*P*-value
CAI Category × Governorate	876.2 (12)	< 0.001
CISI Category × Governorate	1647.4 (12)	< 0.001
API Category × Governorate	1475.7 (12)	< 0.001
BAI Category × Governorate	1237.7 (12)	< 0.001

CAI = Climate Awareness Index; CISI = Climate Impact Severity Index; API = Adaptation Practices Index; BAI = Barriers to Adaptation Index. *df* = degrees of freedom.

The CISI showed the largest regional disparity (χ2 = 1647.4, *P* < 0.001). Menoufia accounted for nearly all high-impact cases, while other governorates (particularly Beheira, Damietta, and Qalyubia) were dominated by lower-impact categories (Fig. 6B).

Variation was similarly noted in the API (χ^2^ = 1475.7, *P* < 0.001). Damietta and Menoufia showed higher adoption levels, whereas Beheira, Kafr El-Sheikh, and Beni Suef exhibited low-to-moderate adoption (Fig. 6C).

Barriers to adaptation (BAI) also differed significantly (χ2 = 1237.7, *P* < 0.001). Beheira exhibited disproportionately high barrier levels, while Minya showed the greatest concentration of low-barrier farmers, reflecting more favorable adaptation conditions in the southern region (Fig. 6D).

Taken together, these results highlight pronounced geographic disparities in climate awareness, experienced climate stress, adaptive capacity, and structural constraints. This underscores the importance of governorate-specific agricultural adaptation policies, rather than uniform national approaches.

### Correlation analysis

**Fig. 7:**
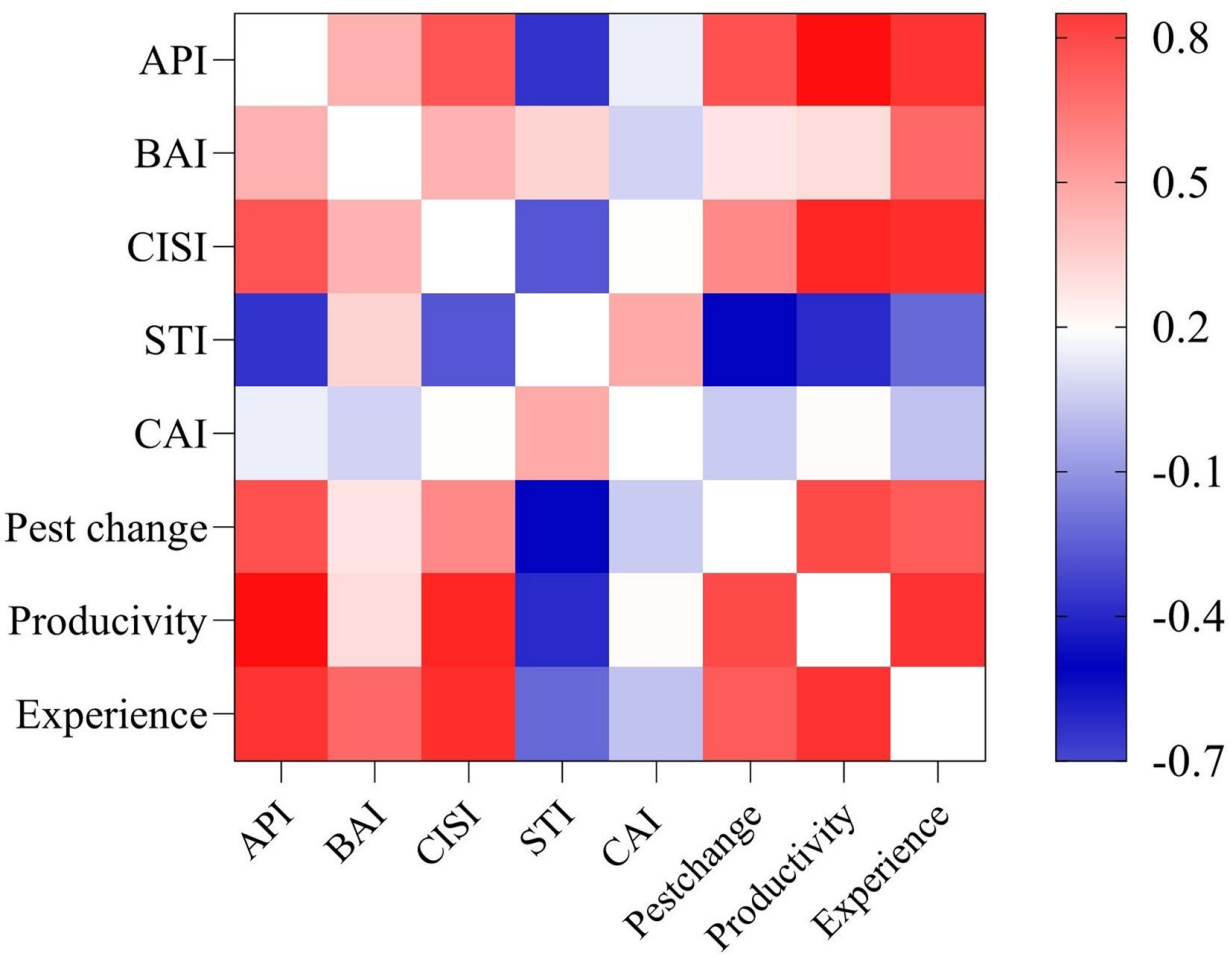
Spearman correlation heatmap of climate–agriculture indices and farm outcomes (N = 2953). Note: CAI = Climate Awareness Index; CISI = Climate Impact Severity Index; API = Adaptation Practices Index; BAI = Barriers to Adaptation Index; STI = Support and Training Index.

Spearman correlation analysis revealed several strong and statistically significant associations among the climate–agriculture indices (Table S1; Fig. 7). The API showed strong positive correlations with productivity change (ρ = 0.80, *P* < 0.01), perceived CISI (ρ = 0.57, *P* < 0.01), and years of farming experience (ρ = 0.68, *P* < 0.01). These relationships indicate that farmers who reported greater climate impacts and had longer farming experience were more likely to adopt a broader range of adaptation practices.

CISI was also strongly associated with changes in productivity (ρ = 0.73, *P* < 0.01) and farming experience (ρ = 0.69, *P* < 0.01). This suggests that farmers perceiving more severe climate impacts were also more likely to report productivity losses and had accumulated greater experience with changing climatic conditions.

The STI demonstrated strong negative correlations with pest-related crop change (ρ =  − 0.69, *P* < 0.01) and productivity change (ρ =  − 0.59, *P* < 0.01). These findings imply that limited access to training or extension support may exacerbate climate-related challenges, including pest pressures and productivity declines.

The BAI’s moderate positive correlation with farming experience (ρ = 0.50, *P* < 0.01) underscores the importance of farmers’ perceptions, encouraging the audience to view their insights as meaningful for understanding adaptation challenges. Meanwhile, the weak correlations between the CAI and other variables (|ρ|< 0.10, *P* < 0.01) suggest that awareness alone is an insufficient driver of behavioral change. Figure 7.

### Multiple regression analysis predicting adaptation practices

A multiple regression analysis was performed to predict farmers’ API scores. The overall model was significant, F(4, 2948) = 1572.83, *P* < 0.001, and explained 68.1% of the variance in API (Table 9). The ANOVA results (Table 10) confirmed the model’s statistical adequacy. Regression coefficients for all predictors are presented in Table 11.

**Table 9:** Model summary for predicting adaptation practices (API).

Metric	Value
R	0.825
R^2^	0.681
Adjusted R^2^	0.680
Std. Error	1.7249
Durbin–Watson	2.026

**Table 10:** ANOVA for regression model predicting API.

Source	SS	df	MS	F	*P*
Regression	18,71861	4	4679.65	1572.83	< 0.001
Residual	8771.21	2948	2.98	–	–
Total	27,489.82	2952	–	–	–

**Table 11:** Coefficients for predictors of adaptation practices (API).

Predictor	B	SE	β	t	*P*	95% CI for B	VIF*
Constant	8.180	0.173	–	47.20	< 0.001	7.84–8.52	–
Change in productivity	2.441	0.072	0.611	33.91	< 0.001	2.30–2.58	3.003
Years of experience	0.643	0.053	0.198	12.07	< 0.001	0.58–0.75	2.479
Crop change due to pests	0.527	0.055	0.136	9.51	< 0.001	0.42–0.64	1.887
CISI	–0.043	0.033	–0.023	–1.28	0.200	– 0.11–0.02	2.993

*Unstandardized (B) and standardized (β) coefficients are reported alongside 95% confidence intervals (CIs) for B. Variance Inflation Factor (VIF) values indicate no severe multicollinearity among predictors (all VIF < 5.0).

Among all predictors, the change in productivity had the strongest positive association with API (β = 0.611, *P* < 0.001, 95% CI: 2.30 to 2.58), indicating that farmers who experienced productivity improvements were substantially more likely to adopt adaptation measures. Years of farming experience (β = 0.198, *P* < 0.001) and crop change due to pests (β = 0.136, *P* < 0.001) also contributed positively. However, CISI was not a significant predictor (*P* = 0.200).

Regression assumptions were evaluated using diagnostic visualizations. The histogram of standardized residuals (Figure S1), the normal P–P plot (Figure S2), and the residual scatterplot (Figure S3) showed acceptable adherence to the assumptions of normality and homoscedasticity. Multicollinearity was not a concern (maximum VIF = 3.00) (Table 11).

## Discussion

This study presents one of the largest farmer-level assessments of climate change perception, agricultural impacts, adaptation behavior, and structural barriers across seven major agricultural governorates in Egypt. The findings uncover a complex yet coherent pathway linking farmer awareness and perceived climatic stressors to agricultural outcomes and adaptive responses, all set within the socioeconomic and institutional realities of Egypt’s agricultural sector.

While farmers demonstrated high levels of climate change awareness, aligning with trends in other developing regions^29,30^, this knowledge failed to translate into meaningful changes in agricultural practices. The weak correlation between the CAI and all agricultural or behavioral measures suggests that simply knowing about climate change is not enough to prompt action. Many farmers clearly recognize the risks they face, yet structural barriers, limited financial resources, and gaps in access to information or support often constrain their ability to respond. This highlights a pronounced “attitude–action gap” driven by systemic bottlenecks. This pattern is well documented in other contexts, where awareness alone rarely leads to adaptation unless farmers also have access to information, extension support, credit, and institutional resources that enable adaptation^10,29,31–34^.

In Egypt specifically, years of direct exposure to extreme heat, rainfall variability, and water scarcity likely heighten awareness. Still, the limited role of governmental extension services and the reliance on peer-to-peer information transfer, as seen in our sample, suggest that awareness is shaped more by direct experience than by formal climate communication systems. This aligns with findings from^35^ and^36^, who emphasize that adaptation failures in smallholder systems are often driven by weak institutional support networks rather than a lack of climate-risk awareness.

Across all governorates, farmers consistently reported increases in rainfall irregularity, temperature rise, and extreme weather events. These perceptions align closely with empirical climatic observations for Egypt and the Middle East and North Africa (MENA) region, which record rising temperatures, increased frequency of heatwaves, and altered precipitation dynamics over the past three decades^37,38^. Studies on farmer perception in similar arid environments confirm that local communities are often highly sensitive to these shifts, accurately tracking meteorological trends through daily experience^36,39,40^.

Yet, when combined within the CISI, most farmers fell into the low-severity category. This seeming contradiction is analytically important because the CISI aggregates four variables into a summed score; many farmers who perceived moderate changes in multiple dimensions did not reach the threshold for high severity. Similar patterns have been documented in climate perception studies in South Asia and Sub-Saharan Africa, where reported experience of climate shocks often diverges from calculated vulnerability scores^41–43^. Nevertheless, the strong correlations among CISI, productivity decline, and years of experience reinforce the idea that farmers with greater exposure to climate events more accurately perceive the severity of climate events and their consequences.

Many farmers reported significant productivity decreases—an alarming figure that mirrors national trends showing yield losses in wheat, maize, rice, and other staples due to warming and water stress^44,45^. The overwhelming perception of pest increases (84.2%) also aligns with climate–pest ecology research showing that higher temperatures accelerate pest reproduction cycles and expand geographic ranges^46^.

The resulting crop changes reported here highlight a fundamental vulnerability: Egypt’s major cereals, especially wheat, are highly exposed to climate-driven pest and heat stress. This presents substantial implications for food security, given the country’s already high reliance on wheat imports.

Despite high awareness and strong climatic stress, adaptation practices were primarily partial and low-level. Only 1.8% of farmers reached a high API score, while 39.2% remained in the low category. The strongest adoption was in traditional climate-smart practices (crop rotation, sustainable inputs), whereas modern technologies, digital tools, and innovative pest management were adopted only minimally. The very low use of digital tools mirrors findings from^47^, which show that adoption of digital agriculture across MENA is constrained by technical literacy, cost, and limited digital extension services.

This deficit is compounded by widespread economic and operational barriers. High input costs, market instability, pest pressures, and skilled labor shortages form the most influential constraints. These findings align with World Bank and peer-reviewed analyses identifying financial liquidity, rising fertilizer prices, and labor scarcity as major constraints on Egyptian agriculture^48–51^. The fact that the BAI correlated moderately with farming experience suggests that more seasoned farmers have a deeper understanding of structural constraints and long-term economic risks. Importantly, uncertainty about the effectiveness of sustainable practices, reported by 33.6% of farmers, reflects a significant gap in extension and knowledge transfer. This mirrors evidence showing that in Egypt, adoption of water-saving, climate-smart, or precision technologies is limited not only by cost but by low trust and poor technical guidance^50,52^.

Nearly half of the respondents reported never receiving adaptation-related training. Weather information services were also minimally used, despite being proven to enhance climate preparedness in other African contexts^53,54^. These deficits create an information-poverty trap, in which farmers perceive risks but lack the technical capacity to respond effectively. The overwhelming demand for awareness programs (84.6%) and preference for governmental extension services underscores a structural opportunity: Egyptian farmers are not resistant to adaptation; they are under-supported.

The remarkably low adoption of modern irrigation (combined 20.4%), despite its critical importance to Egypt’s water security, highlights a fundamental disconnect between national policy goals and on-farm realities^55^. Our findings suggest that barriers to modern irrigation are not merely informational but deeply structural, rooted in high capital costs, land fragmentation, and the complexities of communal water management. To bridge this gap and overcome the “attitude-action” barrier, policy interventions must move beyond general climate awareness campaigns to directly target structural constraints. Actionable strategies should include: (1) providing targeted financial subsidies or microcredit facilities to offset the prohibitive initial costs of modern irrigation and digital tools; (2) revitalizing localized, government-backed extension hubs (such as scaling up Farmer Field Schools) to deliver hands-on, site-specific technical training; and (3) facilitating the development of farmer cooperatives to improve market access, stabilize input prices, and pool resources for mechanized technologies. Ultimately, integrating these farmer-centric support mechanisms into Egypt’s National Climate Change Strategy 2050 is essential to transform reactive, incremental coping strategies into proactive, transformative agricultural resilience.

Regional differences were striking across all indices. Menoufia showed the highest climate impact severity and one of the highest adaptation levels—indicating that farmers in high-stress areas may adopt more practices when feasible. Beheira and Kafr El-Sheikh, large Delta governorates heavily dependent on flood irrigation, showed low awareness and low adaptation, consistent with evidence of slower modernization and high land fragmentation in the Delta region^56–58^. Minya, in Upper Egypt, showed notably lower barriers, suggesting differences in land tenure, crop systems, or institutional support. These patterns emphasize that uniform national adaptation policies are insufficient; instead, regionally tailored strategies are needed to reflect diverse climatic, economic, and institutional realities.

The results reveal clear relationships between climate impacts, adaptation behavior, and agricultural outcomes. The correlation heatmap highlights three notable dynamics. First, higher CISI scores were strongly associated with greater productivity loss, suggesting that farmers who perceive more severe climate impacts are often those experiencing real declines in output. Second, productivity loss showed a strong positive relationship with adaptation uptake, indicating that farmers tend to adopt more adaptation strategies when they face tangible agricultural stress—consistent with reactive rather than anticipatory adaptation. Third, low access to support and training (STI) was associated with both greater pest-related production changes and higher productivity losses, underscoring how limited institutional capacity can intensify vulnerability.

The regression model reinforces these insights by showing that actual productivity change (*β* = 0.611), farming experience, and pest-induced crop changes were the strongest predictors of adaptation behavior. In contrast, perception-based CISI did not significantly predict adaptation after accounting for real stressors. This dynamic confirms that adaptation among smallholders is largely reactive—driven by experienced productivity stress rather than proactive climate awareness^59–62^. These findings correspond with broader empirical evidence showing that experienced climatic shocks, such as yield decline, pest pressure, and rainfall variability, are the primary triggers of adaptation among smallholder farmers^63–65^. This reinforces the conclusion that agricultural stress, rather than climate perceptions alone, is the strongest motivator of behavioral change in smallholder farming systems.

## Conclusion

This study documents a clear pathway from perception to impact to adaptation in Egyptian agriculture. Farmers report high awareness of climate change and experience tangible impacts on crop productivity and pest dynamics; however, adoption of technical adaptation measures remains limited. Adaptive responses are predominantly incremental and shaped by structural and economic conditions rather than awareness alone. These findings highlight the need for region-specific adaptation strategies, improved access to extension services, and wider dissemination of climate-smart agricultural technologies, particularly in highly vulnerable governorates.

The interpretation of these results should be considered within the scope of the study design. The analysis relied on farmer-reported information to capture perceptions, experienced impacts, and adaptation behavior, which is appropriate for examining farm-level decision-making. However, this reliance on self-reported data introduces potential recall and social desirability biases, in which respondents might inadvertently overstate their adaptation efforts or align their answers with perceived expectations. The cross-sectional nature of the survey provides a snapshot of current adaptation patterns, but it restricts our ability to draw definitive causal inferences regarding the directionality between climate impacts and adaptation behavior.

Future research could build on these findings by integrating farmer-reported data with meteorological observations and historical agricultural yield records to triangulate self-reported impacts and minimize bias. Additionally, employing longitudinal study designs would capture the temporal dynamics of adaptation, revealing how farmers’ behaviors evolve over multiple agricultural seasons in response to changing stressors. Finally, experimental approaches, such as randomized controlled trials (RCTs) evaluating specific extension or financial interventions, could robustly identify the causal mechanisms needed to translate climate awareness into effective and durable adaptation.

## Methods

**Fig. 8:**
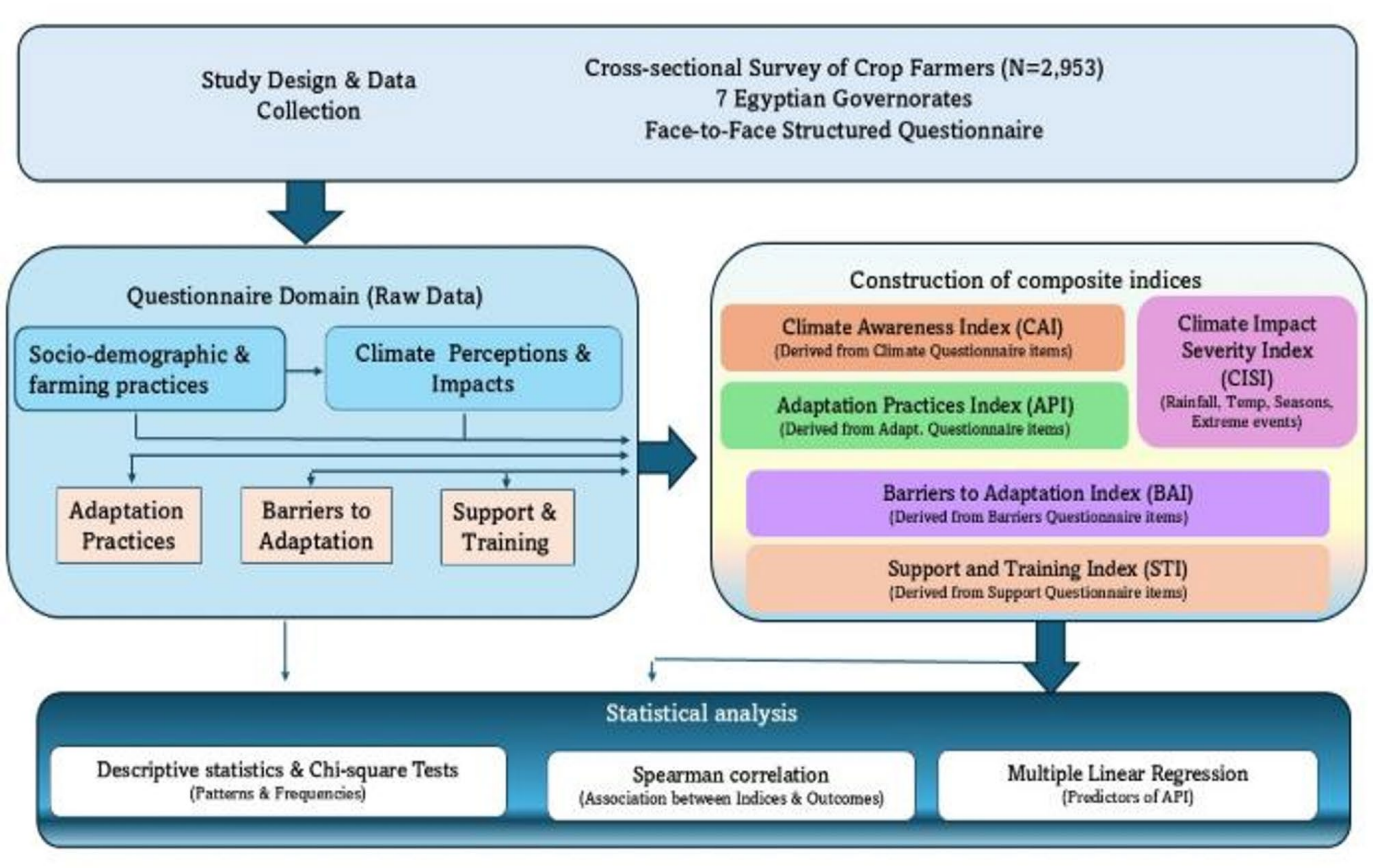
Methodological flow chart illustrates data collection, index construction, and statistical analysis.

An overview of the study design, data collection procedures, index construction, and statistical analyses is presented in Fig. 8.

### Study design and setting

This study employed a cross-sectional survey design targeting crop farmers in seven major agricultural governorates in Egypt: Beheira, Kafr El-Sheikh, Menoufia, Damietta, Qalyubia, Beni Suef, and Minya. Data were collected through structured face-to-face interviews conducted by trained field enumerators. A total of 2,953 farmers provided complete responses and were included in the final analysis.

### Participants

Eligible participants were adult farmers actively cultivating at least one agricultural field within the selected governorates and who self-identified as the primary decision-maker for farm management. Only one respondent per farm holding was interviewed. Participation was voluntary, and all responses were collected anonymously.

### Questionnaire and measures

The questionnaire captured four main domains:

**Socio-demographic characteristics**

Information was collected on age group, education level, years of farming experience, whether farming constituted the main source of household income, number of family members involved in agriculture, land tenure status, and the governorate of residence.

**General farming practices**

Data were collected on irrigation systems, water sources, fertilizer use, level of mechanization, crop rotation practices, and the type and source of seed used.

**Climate change perceptions and impacts**

This domain assessed farmers’ perceived awareness of climate change and its agricultural impacts, as well as their primary sources of climate-related information and perceived changes in rainfall patterns, temperature, agricultural seasons, and extreme weather events. Respondents also reported perceived changes in crop productivity, in the crops most affected by climate variability, in pest pressure, and in pest-attributed crop losses.

**Adaptation practices, barriers, support, and training**

This section captured the adoption of sustainable agricultural practices (*e.g*., soil and water management), irrigation modernization, use of climate-adapted crop varieties, innovative pest and disease management strategies, collaboration with other farmers, and use of digital tools. Barriers to adaptation included perceived major farm challenges, obstacles to adopting sustainable practices, difficulty accessing tolerant seed varieties, marketing constraints, economic pressures, and skilled labor shortages. Support-related items addressed participation in training activities, use of weather forecasts, perceived urgency of awareness-raising, and preferred types and sources of support.

All variables were recorded at the farmer level (N = 2,53). Categorical response options were numerically coded for analysis.

### Construction of composite indices

To summarize key dimensions of climate awareness, perceived climate impacts, adaptation behavior, adaptation barriers, and support systems, five composite indices were constructed. Index development was guided by established conceptual frameworks and informed by prior empirical studies on climate perception, impact severity, adaptation practices, barriers to adaptation, and institutional support mechanisms^24,31,66–77^. To ensure the internal consistency and robustness of the composite indices, Cronbach’s alpha was calculated for each construct prior to aggregation. The reliability analyses yielded acceptable internal consistency for the Adaptation Practices Index (API, α = 0.698) and the Climate Impact Severity Index (CISI, α = 0.654). While the Barriers to Adaptation Index yielded a lower alpha (BAI, α = 0.349)—which is mathematically expected given that the index aggregates independent, multi-dimensional constraints (*e.g*., financial costs vs. technical knowledge) rather than redundant psychological traits—it was retained for its theoretical relevance in capturing cumulative farm-level vulnerability.

### Climate awareness index (CAI)

Climate awareness was assessed using a single item measuring farmers’ perceived awareness of climate change impacts on agriculture. Response categories were coded as: very high awareness = 3, high awareness = 2, and medium or low awareness = 1. This yielded a CAI score ranging from 1 to 3, with higher values indicating greater awareness. For descriptive analyses, CAI was categorized into low (1), medium (2), and high (3) awareness levels. The continuous CAI score was used in correlation and regression analyses.

### Climate impact severity index (CISI)

Perceived climate impact severity was assessed using four items capturing perceived changes in rainfall patterns, temperature, agricultural seasons, and the frequency of extreme weather events. Each item originally included five ordered response categories. Responses were recoded so that higher scores consistently reflected more severe perceived negative climate impacts. The recoded item scores were summed to generate the CISI, with a theoretical range of 4–20. CISI was analyzed as a continuous variable and categorized into low (4–9), moderate (10–14), and high (15–20) impact levels for descriptive purposes.

### Adaptation practices index (API)

The Adaptation Practices Index (API) quantified the extent of climate-related adaptation undertaken by farmers. The index comprised six items capturing sustainable agricultural practices, irrigation modernization, use of climate-adapted varieties, innovative pest and disease management, collaboration with other farmers, and use of digital tools. Each item was rated on a four-point scale ranging from 1 (not adopted) to 4 (fully adopted). Item scores were summed to generate an API score ranging from 6 to 24, with higher values indicating greater adaptation intensity. For descriptive analysis, API values were grouped into low (6–12), moderate (13–18), and high (19–24) adaptation levels, while the continuous score was used for inferential analyses.

### Barriers to adaptation index (BAI)

The Barriers to Adaptation Index (BAI) captured constraints affecting farmers’ ability to implement adaptation measures. Six items assessed major farm challenges, barriers to sustainable adoption, difficulty accessing tolerant seed varieties, frequency of marketing problems, influence of economic factors, and skilled labor shortages. All items were recoded so that higher scores indicated more severe constraints. Summed scores yielded a BAI range of 6–24, with higher values indicating greater barriers to adaptation. For descriptive analyses, BAI values were categorized into low (6–12), moderate (13–18), and high (19–24) barrier levels.

### Support and training index (STI)

Support and training were assessed using two items that captured participation in training or extension activities and the perceived urgency of awareness and support needs. Responses were coded so that higher values reflected greater exposure to training and higher perceived support needs. These items were summed to generate the Support and Training Index (STI), with a theoretical range of 2–8 and an observed range of 3–8. For descriptive analyses, STI was dichotomized into low support (3–4) and adequate support (5–8).

Additional support-related variables (*e.g*., use of weather forecasts, type and source of support) were excluded from index construction due to inconsistent measurement scales and non-monotonic response patterns and were analyzed descriptively.

### Statistical analysis

All analyses were conducted using IBM SPSS Statistics (IBM Corp., Armonk, NY, USA). Categorical variables were summarized using frequencies and percentages, and 95% confidence intervals (CIs) for key estimates to provide a clearer assessment of precision. Continuous indices were summarized using means and standard deviations. Governorate-level differences were examined using cross-tabulations and Pearson’s χ^2^ tests with column proportion comparisons. A two-sided significance level of *P* < 0.05 was applied.

Associations between indices and key climate–agriculture outcomes were examined using Spearman’s rank correlation coefficient (ρ). Correlation results were visualized using a heatmap generated in GraphPad Prism (GraphPad Software, San Diego, CA, USA), with correlations at *P* < 0.01 considered statistically significant.

A multiple linear regression model was fitted with the continuous API score as the dependent variable. Independent variables were selected a priori based on conceptual relevance, theoretical frameworks of agricultural adaptation behavior, and significant bivariate associations to avoid model overfitting. Model assumptions were rigorously assessed using residual diagnostics (including standard residual histograms and normal P-P plots to verify normality and homoscedasticity) and multicollinearity checks (variance inflation factors (VIFs) and tolerance values, with VIFs < 5 deemed acceptable). Statistical significance was set at *P* < 0.05 (two-sided).

### Ethical consideration

The study protocol was reviewed by the Research Ethics Committee of Cairo University, Egypt, which determined that the study did not require formal ethical approval due to its minimal-risk nature and the anonymous collection of data. The study was conducted in accordance with relevant guidelines and regulations and the ethical principles of the Declaration of Helsinki (1964). The committee approved the use of verbal rather than written informed consent. Participation was voluntary, and verbally informed consent was obtained from all participants prior to data collection, confirming their understanding of the study objectives and their right to withdraw at any time.

## Supplementary Information

Below is the link to the electronic supplementary material.

Supplementary Material 1

## Data Availability

The datasets generated and analyzed during the current study are not publicly available due to ethical and confidentiality considerations related to the questionnaire-based survey. Anonymized data may be made available from the corresponding author upon reasonable request.
