# Supplementary material for: Climate change perception–impact–adaptation pathways among farmers: evidence from seven major agricultural governorates in Egypt
Source: Sci Rep. 2026 Jul 29;16:23539. doi: 10.1038/s41598-026-63674-x (PMC13421700; doi:10.1038/s41598-026-63674-x)
Supplement: Supplementary file 1 — Supplementary Material 1 [file 41598_2026_63674_MOESM1_ESM.pdf]

Supplementary data

Climate Change Perception–Impact–Adaptation Pathways Among Farmers: Evidence from Seven Major Agricultural Governorates in Egypt

Eman S. Swelam, Moataz S. Abdallah, Hamed A. Ead, Attia M. El-Tantawi, and Fatma S. Ahmed*

Table S1. Spearman correlations between adaptation practices and key climate–agriculture variables (N = 2,953)

Variable	API	BAI	CISI	STI	CAI	Crop change due to pests	Productivity change	Years of experience
API	1.000	0.260**	0.568**	−0.562**	−0.048**	0.579**	0.797**	0.675**
BAI	0.260**	1.000	0.251**	0.140**	−0.121**	0.085**	0.116**	0.501**
CISI	0.568**	0.251**	1.000	−0.465**	0.002	0.392**	0.726**	0.691**
STI	−0.562**	0.140**	−0.465**	1.000	0.283**	−0.690**	−0.585**	−0.412**
CAI	−0.048**	−0.121**	0.002	0.283**	1.000	−0.143**	0.013	−0.168**
Crop change due to pests	0.579**	0.085**	0.392**	−0.690**	−0.143**	1.000	0.599**	0.542**
Productivity change	0.797**	0.116**	0.726**	−0.585**	0.013	0.599**	1.000	0.680**
Years of experience	0.675**	0.501**	0.691**	−0.412**	−0.168**	0.542**	0.680**	1.000

Note: Index.** $p < 0.01$ (2-tailed) for all correlations.
API = Adaptation Practices Index; BAI = Barriers to Adaptation Index; CISI = Climate Impact Severity; STI = Support & Training Index; CAI = Climate Awareness.

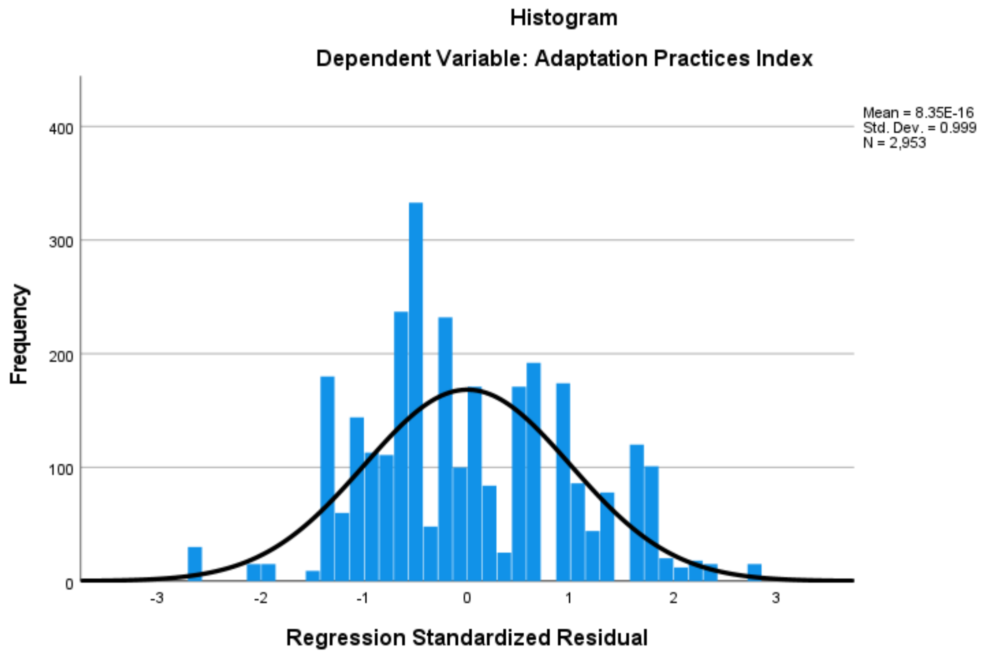

Figure S1. Histogram of standardized residuals showing approximate normality.

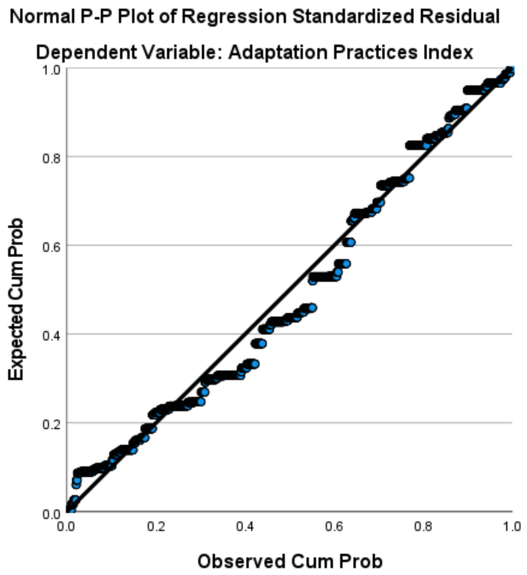

Figure S2. Normal P–P plot demonstrating linear fit between observed and expected residual distributions.

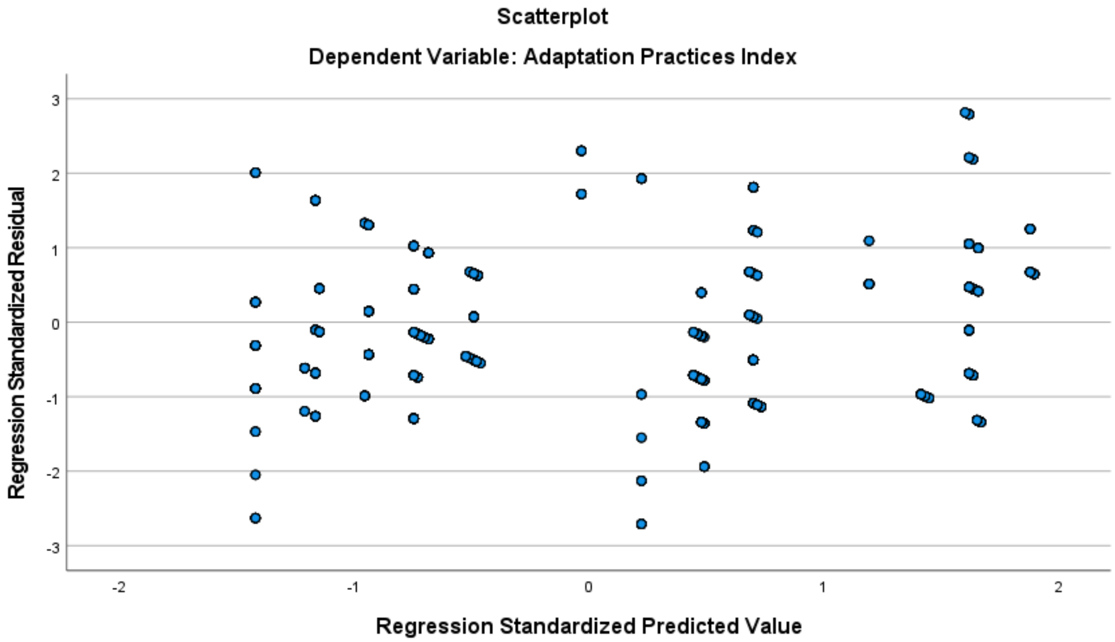

Figure S3. Scatterplot of standardized predicted values vs residuals illustrating homoscedasticity.
